# A genetic programming-based optimal sensor placement for greenhouse monitoring and control

**DOI:** 10.3389/fpls.2023.1152036

**Published:** 2023-06-09

**Authors:** Oladayo S. Ajani, Esther Aboyeji, Rammohan Mallipeddi, Daniel Dooyum Uyeh, Yushin Ha, Tusan Park

**Affiliations:** ^1^ Department of Artificial Intelligence, School of Convergence, Kyungpook National University, Daegu, Republic of Korea; ^2^ Department of Biosystems and Agricultural Engineering, Michigan State University, East Lansing, MI, United States; ^3^ Upland-Field Machinery Research Center, Kyungpook National University, Daegu, Republic of Korea; ^4^ Department of Bio-Industrial Machinery Engineering, Kyungpook National University, Daegu, Republic of Korea; ^5^ Smart Agriculture Innovation Center, Kyungpook National University, Daegu, Republic of Korea

**Keywords:** sensor aggregation, optimal sensor location, genetic programming, greenhouse, control

## Abstract

Optimal sensor location methods are crucial to realize a sensor profile that achieves pre-defined performance criteria as well as minimum cost. In recent times, indoor cultivation systems have leveraged on optimal sensor location schemes for effective monitoring at minimum cost. Although the goal of monitoring in indoor cultivation system is to facilitate efficient control, most of the previously proposed methods are ill-posed as they do not approach optimal sensor location from a control perspective. Therefore in this work, a genetic programming-based optimal sensor placement for greenhouse monitoring and control is presented from a control perspective. Starting with a reference micro-climate condition (temperature and relative humidity) obtained by aggregating measurements from 56 dual sensors distributed within a greenhouse, we show that genetic programming can be used to select a minimum number of sensor locations as well as a symbolic representation of how to aggregate them to efficiently estimate the reference measurements from the 56 sensors. The results presented in terms of Pearson’s correlation coefficient (*r*) and three error-related metrics demonstrate that the proposed model achieves an average *r* of 0.999 for both temperature and humidity and an average RMSE value of 0.0822 and 0.2534 for temperate and relative humidity respectively. Conclusively, the resulting models make use of only eight (8) sensors, indicating that only eight (8) are required to facilitate the efficient monitoring and control of the greenhouse facility.

## 1 Introduction

Optimal sensor placement is aimed at realizing a sensor profile or layout that achieves minimum cost as well as satisfies some pre-specified performance criteria has gained traction in a broad spectrum of applications areas such as health monitoring (Tan and Zhang, 2020), distribution of medicine in disaster areas (Parque et al., 2019), indoor cultivation systems (Uyeh et al., 2022b) and smart cities (Du et al., 2019; Jena et al., 2021). Specifically, in indoor cultivation systems, optimal sensor placement has become attractive to facilitate the efficient coordination of sensors for monitoring plant life as well as providing the necessary control of the internal environmental conditions (micro-climate). Indoor cultivation systems such as greenhouses are cultivation systems that are controlled in order to support all year-round growing of plants or crops (Nordey et al., 2017). Although these systems are economical compared with open field cultivation systems, they rely on effective monitoring and control of micro-climate such as temperature and humidity which have a direct impact on crop growth, quality (Takahata and Miura, 2017; Syed and Hachem, 2019) and consequently, crop yield (Nordey et al., 2017). In fact, experimental analysis has shown that while effective control of the temperature favors plant growth and reduces the overall energy consumption of the system, appropriate levels of relative humidity are necessary to prevent fungal infections and control transpiration (Vox et al., 2010). In other words, efficient monitoring and control of micro-climate are crucial to achieving the economic and sustainability goals of controlled cultivation systems.

Traditionally, monitoring of greenhouse micro-climate and consequently its control is facilitated through randomly distributed sensors (based on the available resources and size of the greenhouse) (Yeon Lee et al., 2019). However, under such settings, there is no guarantee that such randomly placed sensors would provide measurements that are representative of the true micro-climatic conditions of the greenhouse. Furthermore, the use of a large number of sensors results in a large amount of data that requires efficient data management. In other words, the quality of information and the accuracy of the resulting micro-climate heavily relies on the number of sensors and their locations/placements. Therefore, the non-trivial task of optimizing the number of sensors and their locations becomes eminent as it forms the basis for accurate measurement of micro-climate and consequently optimal control of the cultivation system. Additionally, it reduces the overall operating cost of controlled cultivation systems.

Although several techniques (Kubrusly and Malebranche, 1985; Alonso et al., 2004; Flynn and Todd, 2010; Yi et al., 2011) for optimal sensor placement have been proposed in the literature for different applications, some of the proposed methods are not directly applicable for highly non-linear setups (complex systems) such as controlled cultivation systems. In the context of controlled cultivation systems, optimization, and machine learning-based algorithms have been proposed (Yeon Lee et al., 2019; Wu et al., 2020; Uyeh et al., 2021; Uyeh et al., 2022b).

In Yeon Lee et al. (2019), a setup which relies on the fusion of an error-based and entropy-based method was proposed for optimal location of temperature sensors. In the setup, a reference temperature is generated by averaging the temperature data from all the measurement locations. Consequently, sensor locations with measurements that are statistically close to reference temperature were selected. In addition, entropy related information was used to select locations that are significantly influenced by external environmental conditions. Based on these two methods, optimal sensor locations that provide representative data of the entire greenhouse condition as well as understanding regions with high variations in temperature were realized. A hierarchical cooperative particle swarm algorithm was proposed in Wu et al. (2020) for sensor placement in a vegetable-cultivating greenhouse with the aim of maximizing the entire coverage area (i.e., a non-occlusion coverage scheme). In the scheme, the decision space was designed based on the global effective coverage of each sensor as well as the orientation angles of the respective sensors. Based on the results, the model was argued to demonstrate the capability to overcome issues of occlusion between covered objects and also improved sensor utilization in general. However, the aforementioned works are limited because they were investigated over a limited period of time which does not account for different planting seasons and weather conditions. To address these issues, (Uyeh et al., 2021) proposed a Reinforcement Learning (RL) based method to optimally place sensors in a greenhouses using a robust dataset which features different planting seasons. The dataset consists data from 56 dual temperature and humidity sensors distributed within a greenhouse. In the work, RL-based ranking of the sensor locations was performed in order of their importance in estimating the greenhouse micro-climate for temperature and relative humidity respectively. The results show that the rank of each sensor location for effective measurement of the greenhouse micro-climate varies from month to month. This is very intuitive because it is expected that different temperature and humidity profiles would occur in different months and/or planting seasons based on the changes in external weather conditions. Based on the same dataset and extracted psychrometric features (dew point temperature, enthalpy, humid ratio, and specific volume) (Uyeh et al., 2022a) proposed a machine learning-based sensors clustering system to find the optimal sensor locations. The results indicate that less than 10 percent of the sensors were required to facilitate effective monitoring of the greenhouse.

Although the aforementioned works have considered optimal sensor location in controlled cultivation system over different planting seasons and environmental conditions, it is important to realize that the ultimate goal of monitoring in controlled cultivation systems is to maintain or regulate the micro-climates to be within the desired range and this is facilitated through the associated control systems. However, these works have only considered the problem of optimal sensor placement from a monitoring or measurement perspective without any notion of control. Therefore deviating from the large body of previous works, this paper proposes a Genetic Programming (GP)-based optimal sensor placement from a control perspective for controlled cultivation systems. In the approach, firstly, we show that reference micro-climate obtained from the aggregation of all measurements from the 56 sensors is highly correlated to measurements from each of the sensors. This means that the reference temperature is a robust estimate of the overall micro-climatic condition of the greenhouse. This is important because, in terms of regulating the micro-climate within the greenhouse, only such reference micro-climate which are representative of the entire environmental condition are required to serve as input to the dedicated control systems. Consequently, reference micro climate obtained based on the weighted averaging aggregation method are used as targets to fit GP models that can effectively model the reference micro-climate using only measurements from sensors that are most vital to the reference micro-climate. In other words, through an optimization process, GP selects only the crucial sensors and effectively fuses them to realize the reference micro-climate. Therefore, the locations of the sensor that are featured in the resulting GP model are the optimal sensor locations required to facilitate monitoring and control of the entire greenhouse. Consistent with the findings in Uyeh et al. (2021), the results show that different optimal sensor locations are representative of the entire environmental condition across different months and different micro-climate. Furthermore, the economic impact of the results is reflected in the observation that only eight (8) sensors are required to monitor and control the controlled cultivation system. This implies that the energy cost of running the greenhouse as well as the sensor procurement cost is reduced drastically.

The rest of the paper is structured as follows; Section II presents a description of the data and featured pre-processing. Furthermore, an overview of data aggregation and the methods employed in this work, as well as correlation analysis of the resulting reference micro-climate compared to the measurements from each of the 56 sensors is presented. In Section III, the background of Genetic Programming as well as the proposed modules are presented. Section IV presents the results in terms of the models obtained as well as their implications. In Section V, conclusions and future directions are highlighted.

## 2 Data description and aggregation

**Figure 1:**
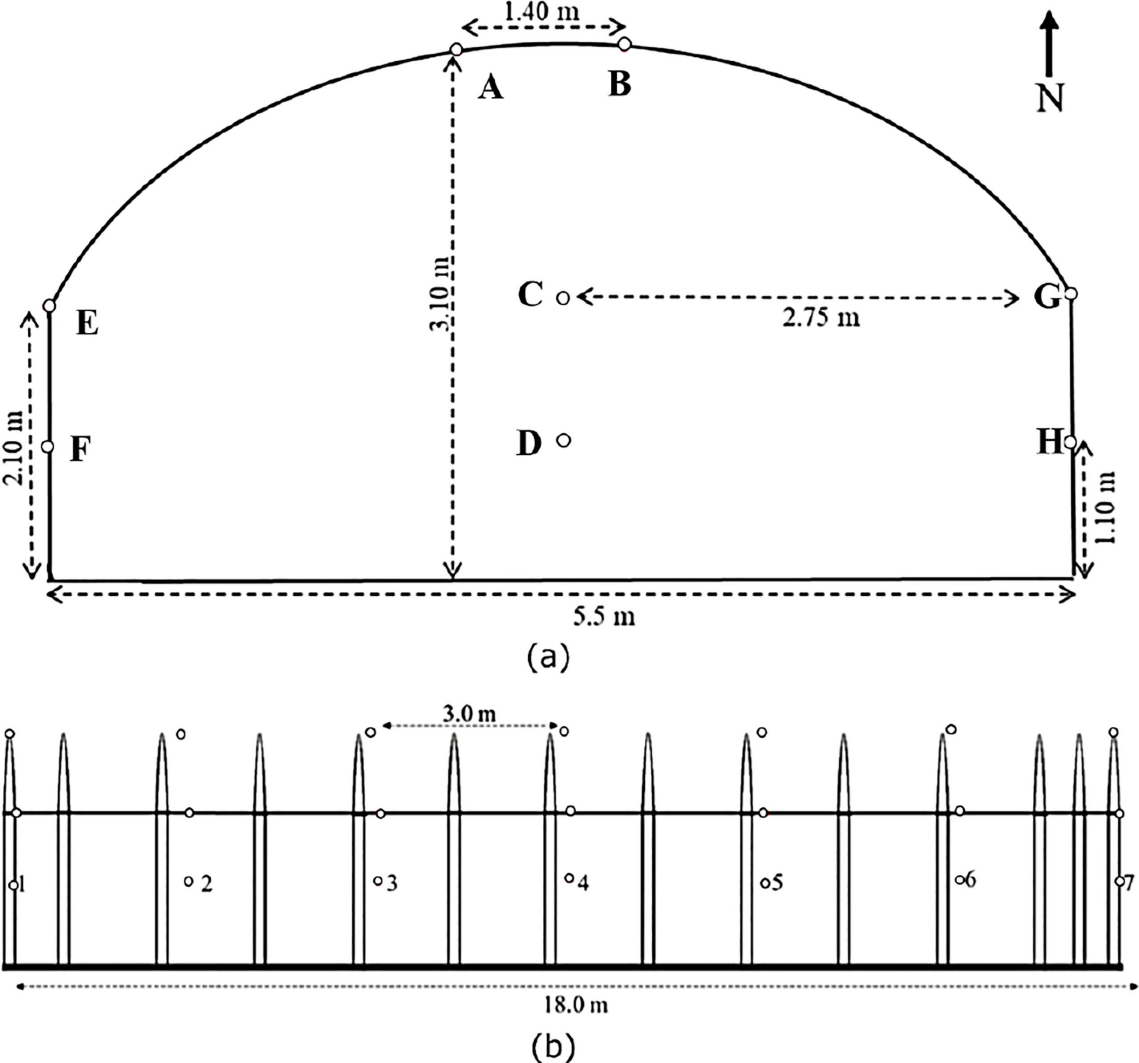
Layout of the 56 two-in-one temperature and relative humidity sensors within the greenhouse **(A)** Front view, **(B)** Side view. The small circles represent the positions of each of the sensors distributed over 8 rows (A–H) with each row containing 7 sensors.

This work leverages on the same data used in (Uyeh et al., 2021; Uyeh et al., 2022a). The dataset contains temperature and relative humidity measurements collected remotely from a research cultivation-controlled system in Kyungpook National University, South Korea. The data was collected over a period of seven months (February, March, April, May, June, July, and October) using 56 dual temperature and relative humidity sensors carefully distributed within the greenhouse. Specifics about the site location, description, greenhouse layout and the data collection protocol are detailed in (Uyeh et al., 2021; Uyeh et al., 2022a). **Figure 1** presents the layout that is representative of the location of each of the sensors within the greenhouse.

The resulting data includes measurements recorded per minute for the two micro-climate (temperature and relative humidity). In terms of pre-processing, rows with missing data points were removed as the number of rows with missing data points is very insignificant compared with the entire observations.

### 2.1 Data aggregation

In the context of achieving a controlled or regulated environment, aggregate micro-climate (relative humidity and temperature) are required as inputs to dedicated control systems for appropriate control actions within the cultivation systems (Yeon Lee et al., 2019). Data aggregation is the process of fusing information from different or multiple sensors together in order to derive a single reference measurement that is sent to a base station or controller depending on the intended application (Al-kahtani and Karim, 2018; Kaur and Munjal, 2020; Yuan et al., 2021). Generally, in controlled cultivation systems the aim is to ensure that the micro-climate are controlled to support plant life and growth. To facilitate such control, there is need to have reference micro-climate that is representative of the environmental conditions of the cultivation systems and consequently take control actions based on the associated control laws. Several data aggregation methods such as weighted averaging (Hang et al., 2017; Yeon Lee et al., 2019), median (Cocco et al., 2015) and more complex fusing algorithms such as the unscented Kalman filter (Xia et al., 2022) and weighted least square method (Ren et al., 2017) etc. have been proposed in the literature for application in cultivating systems and other application domains.

In this work, we use the simple weighted averaging method given as


(1) $$W=\frac{\sum_{i=1}^{N}w_{i}X_{i}}{\sum_{i=1}^{N}w_{i}}$$

where *N* is the total number of sensors to be averaged, $w_{i}$ is the weights applied to each sensor value and $X_{i}$ is the sensor values to be averaged. Similar to (Yeon Lee et al., 2019), we take the weight $w_{i}=1$ for all the 56 sensors. This is to ensure that every variation or section of the greenhouse is given equal important. Furthermore, to ensure that the chosen aggregation method is representative of the response of each sensor we perform correlation analysis of the reference micro-climate with micro-climate from each of the sensors.

## 3 Genetic programming-based optimal sensor location

In this Section, a systemic overview of GP is presented and consequently, the protocols of the GP for the optimal sensor location based on the aforementioned data are presented.

### 3.1 Genetic programming

In artificial intelligence, Genetic programming (GP) is a class of bio-inspired algorithms generally known as evolutionary algorithms that are capable of generating solutions to problems that humans cannot solve or do not know how to solve directly. Formally, GP is a systematic method for getting computers to automatically solve a problem starting from a high-level statement of what needs to be done (Koza and Poli, 2005). Generally, based on different genetic operations (genetic events) such as crossover, mutation, reproduction, gene duplication, and gene deletion the idea is to randomly generate a large set of solutions and to evolve those solutions until the population converges to a global maxima/minima depending on the associated task and termination criteria. It is often used in the field of Machine Learning for hyper-parameter selection (Agrawal et al., 2021) or to determine relationships between features in data (Rodrigues et al., 2022). For example in the context of this work, the measurements from the 56 sensors are features and we intend to select the best features corresponding to the optimal sensor locations.

In terms of implementation, the typical evolution process of GP involves the following steps:

1. Define the problem objectives and randomly initialize or generate a population of solution candidates.
2. Repeat the following steps until a pre-defined termination criterion is reached:
  (a) Evaluate each of the solution candidates in the population based on the problem objective and assign it a function value.
  (b) Generate a new population of solution candidates by performing the following operations:
    1) Select a set of solution candidates for mating based on the assigned fitness value (selection).
    2) Include some of the selected solution candidates into the new population without modifying them (reproduction).
    3) Generate new solution candidates by genetically recombining randomly chosen parts of two selected individuals (crossover).
    4) Generate new solution candidates by replacing randomly chosen parts of some selected individuals with new randomly generated ones (mutation).
3. The resulting best solution candidates at any generation of the evolution process is chosen as the result of the GP process.

**Figure 2:**
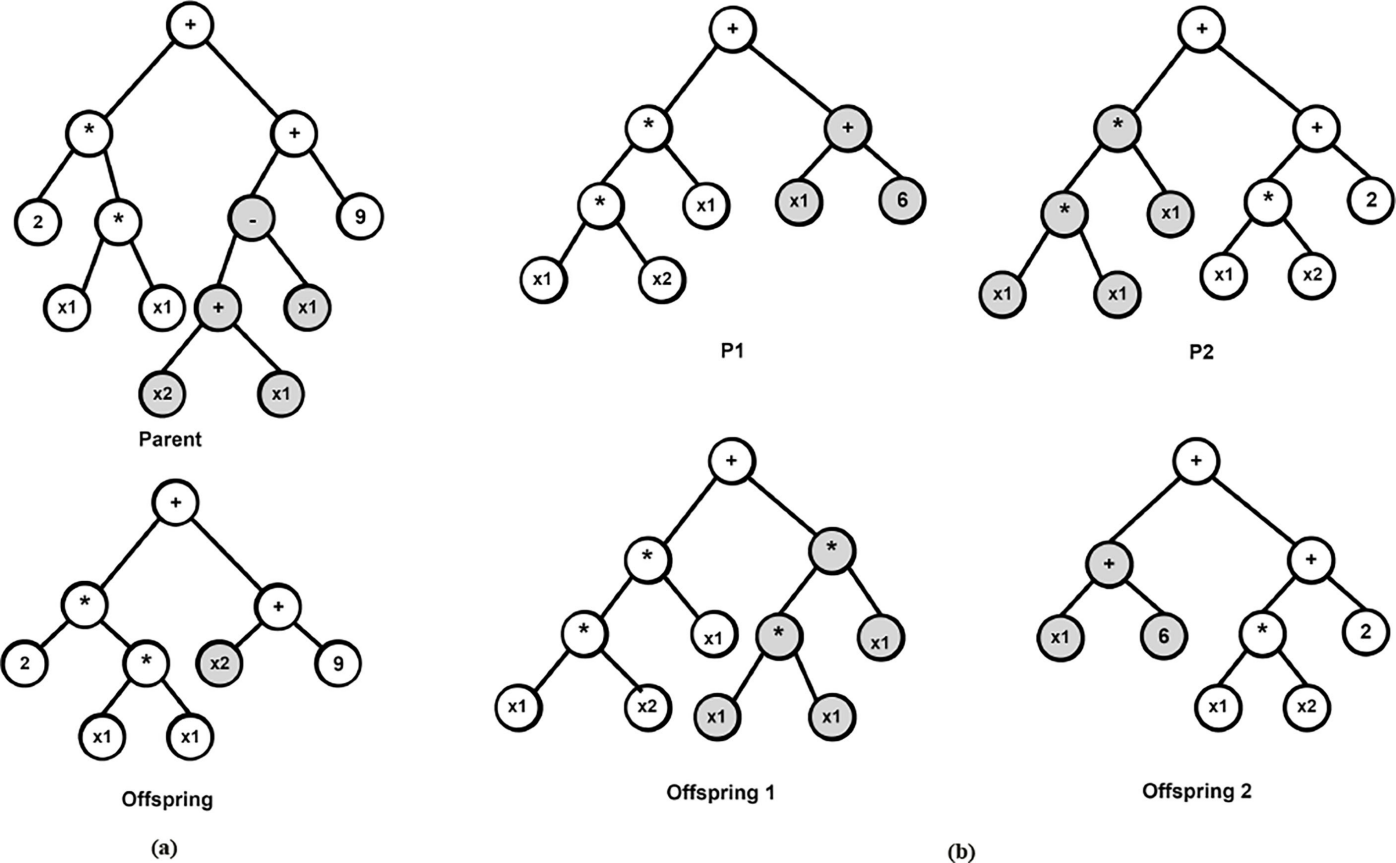
Examples of mutation and crossover operations in GP **(A)** Offspring generated by a single-parent mutation **(B)** Two offspring are generated by a bi-parent crossover.

The aforementioned steps are summarized mathematically in Algorithm 1. In classical GP, solution candidates or programs are encoded as tree-based structures as shown in **Figure 2** (Koza, 1993) because evaluating trees in a recursive manner is easy. Under this setting, mathematical expressions are evolved and evaluated with each tree nodes having an operator function and each terminal mode an operand. Furthermore, the crossover operation is achieved by swapping randomly selected sub-trees from two parent candidates while mutation is achieved by replacing a randomly chosen individual’s sub-tree by a randomly generated one (Sotto et al., 2021). For example, in **Figure 2A**, the sub-tree of the parent solution is replaced to produce the offspring and in **Figure 2B**, two parents P1 and P2 are crossed to produced offspring 1 and 2 accordingly.

### 3.2 GP-based optimal sensor location

In order to evolve a GP model that is representative of the optimal sensor locations, it’s important to set the global task or objective. In terms of optimal sensor location, the goal is to realize an efficient combination of a limited number of sensors that can estimate the reference micro-climate obtained from the aggregation of the 56 sensors. Since the reference micro-climate are real continuous values, the problem at hand can be formalized as a classical symbolic regression task which is one of the most widely studied application of GP (Uy et al., 2010; He et al., 2022; Zojaji et al., 2022). Symbolic Regression (SR) is a class of machine learning approach that searches the space of mathematical expressions with the aim of identifying a model or expression that best describes the relations between a given dataset, both in terms of accuracy and simplicity. This can be summarized as a multi-objective framework where accuracy (error) is maximized (minimized) and the number of sensors is minimized (simplicity). Therefore, given the measurements of 56 sensors as input for each of the associated micro-climate and a set of operator functions, the GP builds a symbolic regression model and selects the minimum number of sensors sufficient to estimate the reference extcolor micro-climate from the 56 sensors. Consequently, the resulting locations of the chosen sensors are the optimal sensor locations for the associated micro-climate and month.

## 4 Computational experiments

Based on the data collected for each of the month featured in the aforementioned dataset, we construct GP models using variables (data from each of the sensors) as well as random numbers as terminals and arithmetic operators such as (addition, multiplication etc.) as operator functions. The choice of constructing GP models based on each month was motivated by intuition that different sensor profiles would be optimal for different months and seasons which was also validated in (Uyeh et al., 2021). For each month and the associated micro-climate, the data is divided randomly into training and testing set based on 70:30 ratio. Furthermore, the training set is further divided to obtain a validation set based on 80:20 ratio. The random division of the data is chosen to ensure that the opportunity to model the different time trends is not missed. Because the validation of the model would be affected if certain time trends are ignored in model development.

All the experiments were conducted in MATLAB installed on a 64-bit Windows 11 PC, with 3.00GHz Intel-i5-12500 CPU and 32GB RAM. The GP is initialized with a population size of 500 and is allowed to evolve for 100 generations. The best results obtained over 25 independent runs of the GP algorithm are reported. In terms of selection, tournament selection (Fang and Li, 2010) with size of 25 was used and an elite fraction of 0.3. For all the experiments, the set of function nodes used are basic arithmetic operators (+, -, ×) as well as minimum (min) and maximum (max) operators.

## 5 Results and discussion

To evaluate the resulting GP models, we employ a number of metrics namely; Pearson’s Correlation Coefficient (R), Root Mean Squared Error (RMSE), Mean Average Error (MAE) and Maximum Absolute Error (Max.AE).

### 5.1 Correlation of sensor aggregation

**Table 1** shows the average correlation of the reference micro-climate (temperature and relative humidity) with each of the measurements from the 56 sensors over the even months. As seen in **Table 1**, the reference textcolormicro-climate are highly correlated with those measured from each of the sensors with the lowest being 97%. This demonstrates that the reference micro-climate based on the average aggregation method is satisfactorily representative of the global environmental conditions of the controlled cultivation systems and can be used to facilitate the control of the entire regions of the cultivation system.

**Table 1:** Average correlation of the reference micro-climate with each of the measurements from the 56 sensors.

Months	Correlation Coefficients (*r*)	
	Temperature	Relative humidity
February	0.972	0.978
March	0.973	0.978
April	0.980	0.980
May	0.980	0.977
June	0.976	0.984
July	0.977	0.985
October	0.970	0.983

### 5.2 Performance of GP-based model for temperature

**Figure 3:**
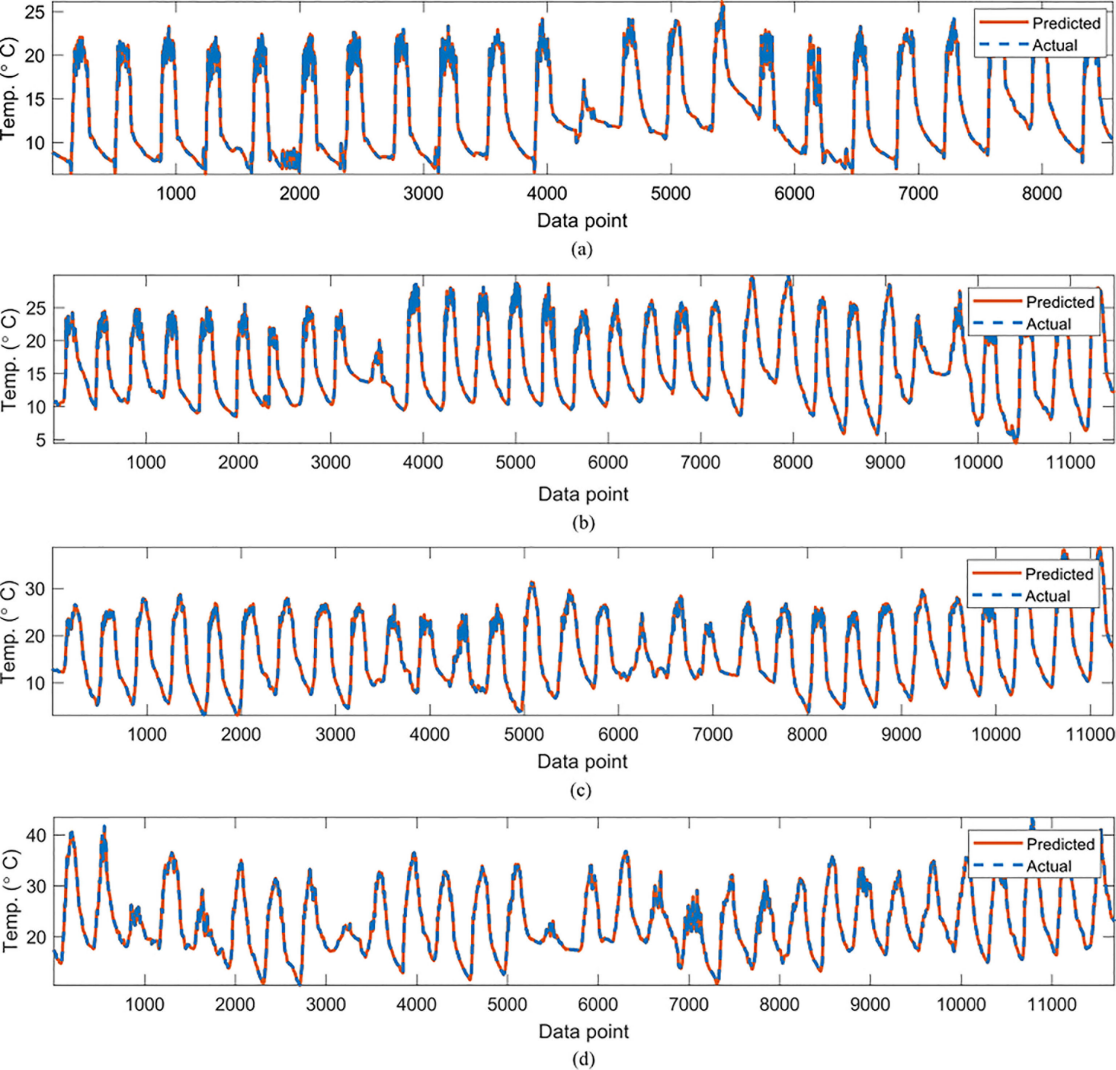
Comparisons of the actual reference temperature versus those predicted by the proposed GP-models for **(A)**February, **(B)** March, **(C)**, April **(D)** May.

**Figure 4:**
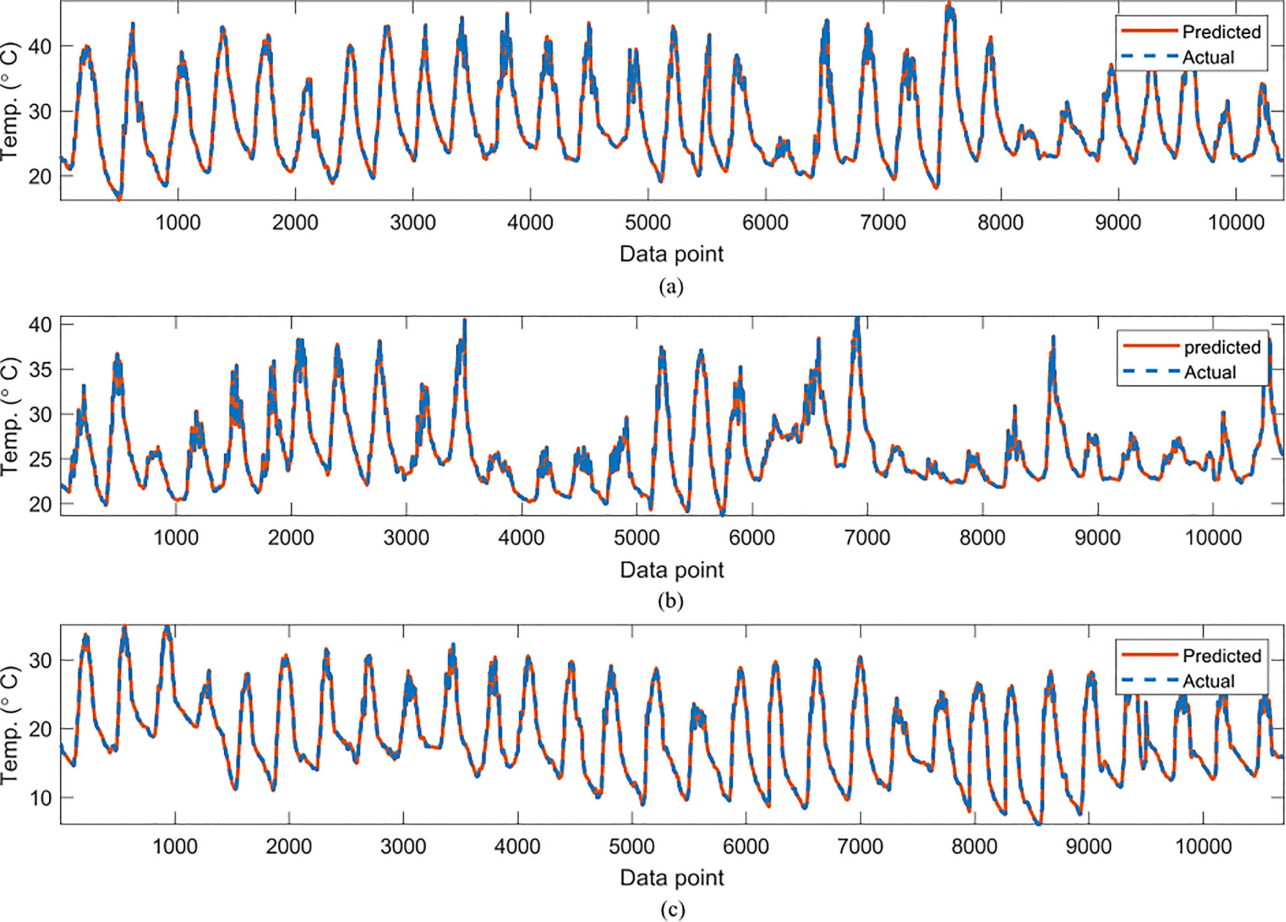
Comparisons of the actual reference temperature versus those predicted by the proposed GP-models for **(A)** June, **(B)** July **(C)** October.

In **Table 2**, the symbolic equations for the resulting model based on the associated sensors are presented. Specifically, the equations represent how to aggregate the information from each sensor as well as the bias term. Based on those models, **Table 3** presents the performance of the model in terms of Pearson’s Correlation Coefficient (*r*) with the reference temperatures, Root Mean Squared Error (RMSE), Mean Average Error (MAE) and the Maximum Absolute Error (Max.AE) of the predicted temperature compared to the reference temperature for each of the 7 months. In **Figures 3**, **4**, comparisons between the actual and predicted values based on the test dataset are presented. The results in terms of the *r* values shows that the actual and predicted temperature based on the GP model are highly correlated with an average value of over 0.99 across the seven months. In terms of the error-related metrics, such as RMSE and MAE, it can be seen from **Table 3** that the values are insignificant and within allowable limits. It is important to note that those error values are not from normalized samples but are based on the real magnitudes of the temperature measurements. The Max.AE metric presents the worst cases of error between the actual and real temperature values. These values are found to be in the region of the allowable measurement error from the device manufacturer which is ± 0.3°C (Uyeh et al., 2021). In terms of the qualitative analysis of the actual and predicted temperature values presented in **Figures 3**, **4**, it can be clearly seen that the actual and predicted temperature measures are very similar.

**Table 2:** Resulting GP-based symbolic models (equations) for temperature.

Months	Symbolic Equations for Temperature
February	0.126A1 + 0.126A2 + 0.126B5 + 0.126B6 + 0.126D7 + 0.126E4 + 0.126F4 + 0.126H1 - 0.1880
March	0.126A1 + 0.126A2 + 0.126C5 + 0.126C7 + 0.126D5 + 0.126E2 + 0.126F5 + 0.126G3 - 0.0887
April	0.125A4 + 0.125B3 + 0.125B5 + 0.125C1 + 0.125D6 + 0.125E1 + 0.125E4 + 0.125F5 - 0.0672
May	0.125A5 + 0.125C1 + 0.125D4 + 0.125E4 + 0.125E6 + 0.125F3 + 0.125G5 + 0.125H1 - 0.0295
June	0.124B1 + 0.124B5 + 0.124B7 + 0.124D5 + 0.124E4 + 0.124E6 + 0.124F3 + 0.124H1 + 0.1330
July	0.125B3 + 0.125B4 + 0.125C7 + 0.125E4 + 0.125E6 + 0.125F5 + 0.125G4 + 0.125H1 + 0.0312
October	0.125B2 + 0.125B7 + 0.125D5 + 0.125E6 + 0.125F2 + 0.125G5 + 0.125H1 + 0.125H3 - 0.0088

**Table 3:** Performance of the GP-based models in terms of Pearson’s Correlation Coefficient (*r*) with the reference temperature, Root Mean Squared Error (RMSE), Mean Average Error (MAE) and the Maximum Absolute Error of the predicted temperature (Max.AE).

Metrics	Months						
	February	March	April	May	June	July	October
*r*	0.9997	0.9998	0.9996	0.9999	0.9999	0.9999	0.9998
RMSE	0.0884	0.0862	0.1490	0.0761	0.0601	0.03178	0.0836
MAE	0.0634	0.0502	0.0644	0.0442	0.0383	0.0210	0.0527
Max.AE	0.4842	0.5808	1.1346	0.5672	0.4539	0.2613	0.6350

### 5.3 Performance of GP-based model for relative humidity

**Figure 5:**
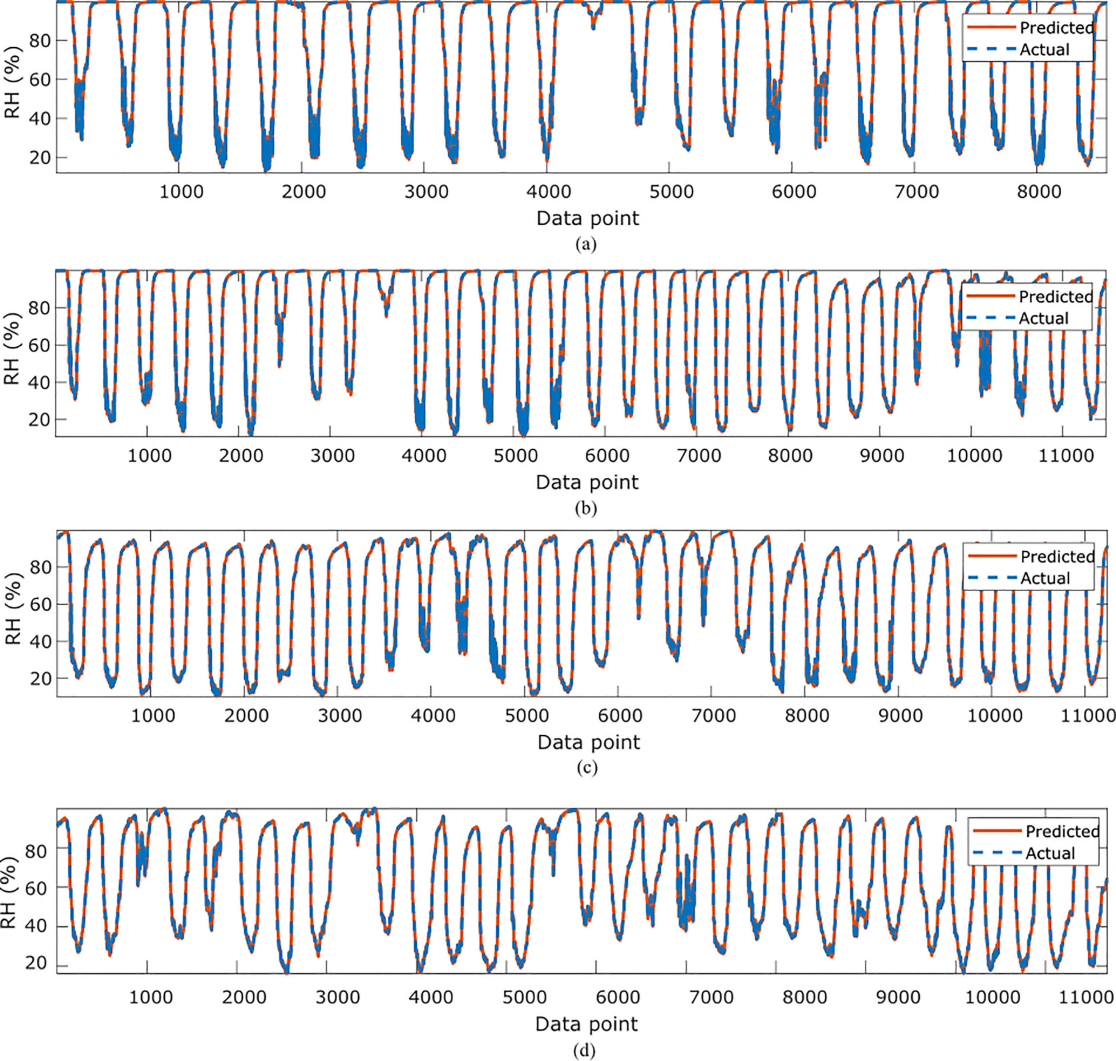
Comparisons of the actual relative humidity versus those predicted by the proposed GP-models for **(A)**February, **(B)** March, **(C)**, April **(D)** May.

**Figure 6:**
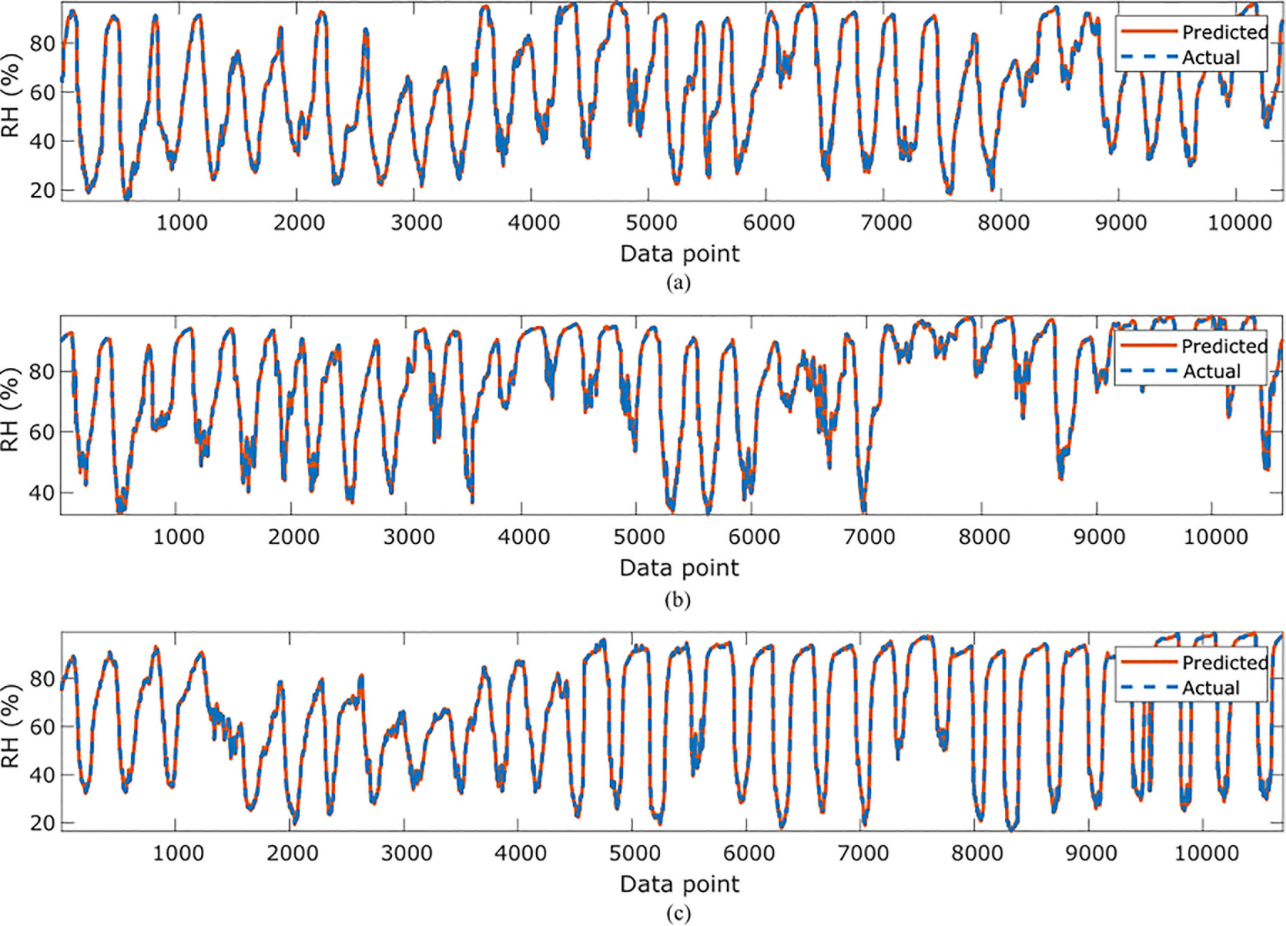
Comparisons of the actual relative humidity versus those predicted by the proposed GP-models for **(A)** June, **(B)** July **(C)** October.

In **Table 4**, the symbolic equations for the resulting model based on the associated sensors are presented. Based on those models, **Table 5** presents the performance of the model in terms of Pearson’s Correlation Coefficient (*r*) with the reference relative humidity, Root Mean Squared Error (RMSE), Mean Average Error (MAE) and the Maximum Absolute Error (Max.AE) of the predicted relative humidity compared to the reference relative humidity for each of the 7 months. In **Figures 5**, **6**, comparison between the actual and predicted values based on the test dataset are presented. The results of the correlation analysis presented in **Table 5** shows that the actual and predicted relative humidity based on the GP-model are highly correlated with an average value of over 0.99 across the seven months. In terms of the error related metrics, such as RMSE and MAE, it can be seen from **Table 5** that the values are insignificant and within allowable limits. It is important to note that those error values are not from normalized samples but are based on the real magnitudes of the relative humidity measurements. The Max. AE metric presents the worst cases of the error between the actual and real relative humidity values. These values are found to be in the region of the allowable measurement error from the device manufacturer which is ± 2%°C (Uyeh et al., 2021). In terms of the qualitative analysis of the actual and predicted relative humidity values presented in **Figures 5**, **6**, it can be clearly seen that the actual and predicted relative humidity values are very similar.

**Table 4:** Resulting GP-based symbolic models (equations) for humidity.

Months	Symbolic Equations for Humidity
February	0.126A1 + 0.126A3 + 0.126A5 + 0.126B6 + 0.126D6 + 0.126E2 + 0.126F4 + 0.126G3 - 0.8330
March	0.125A1 + 0.125A4 + 0.125B7 + 0.125C5 + 0.125D3 + 0.125E1 + 0.125E6 + 0.125F3 + 0.0494
April	0.126A1 + 0.126A4 + 0.126A5 + 0.126B5 + 0.126B6 + 0.126C3 + 0.126F6 + 0.126H1 - 0.6660
May	0.125B4 + 0.125B6 + 0.125C1 + 0.125C2 + 0.125C3 + 0.125D7 + 0.125F6 + 0.125H1 - 0.1570
June	0.125B6 + 0.125B7 + 0.125C1 + 0.125C3 + 0.125D3 + 0.125D7 + 0.125F2 + 0.125G4 - 0.0069
July	0.124B2 + 0.124B7 + 0.124C1 + 0.124C4 + 0.124D6 + 0.124E2 + 0.124E6 + 0.124H3 + 0.4730
October	0.125A4 + 0.125B6 + 0.125B7 + 0.125C3 + 0.125E1 + 0.125E2 + 0.125F5 + 0.125H5 - 0.2610

**Table 5:** Performance of the GP-based models in terms of Pearson’s Correlation Coefficient (*r*) with the reference relative humidity, Root Mean Squared Error (RMSE), Mean Average Error (MAE) and the Maximum Absolute Error (Max.AE) of the predicted relative humidity.

Metrics	Months						
	February	March	April	May	June	July	October
*r*	0.9999	0.9999	0.9999	0.9999	0.9999	0.9999	0.9999
RMSE	0.3397	0.3152	0.2943	0.2196	0.2115	0.1536	0.2399
MAE	0.1786	0.1864	0.1722	0.1374	0.1462	0.1215	0.1659
Max.AE	4.1358	4.2093	4.1959	3.1712	2.2670	1.0564	3.4247

### 5.4 Analysis of selected optimal sensor locations

In **Table 6**, the selected optimal sensor locations for each month are presented for temperature. From the Table, it can be observed that for the months of February, March and April an average of 5 out of the eight sensors selected are distributed along the center of the greenhouse (*A, B, C, D*), while other remaining three are either to the right or left side of the greenhouse. On the other hand for May to October, it can be seen that only an average of 3 of the sensors selected are distributed along the center of the greenhouse while the others are distributed to the left or right side of the greenhouse. This can be attributed to the different seasons of each month. Specifically, it can be inferred that the colder months have higher concentration of sensors along the center of the greenhouse while the more hotter months takes more advantage of sensor distributed along the facility.

**Table 6:** Optimal sensor locations for temperature.

Optimal sensor locations						
February	March	April	May	June	July	October
A1	A1	A4	A5	B1	B3	B2
A2	A2	B3	C1	B5	B4	B7
B5	C5	B5	D4	B7	C7	D5
B6	C7	C1	E4	D5	E4	E6
D7	D5	D6	E6	E4	E6	F2
E4	E2	E1	F3	E6	F5	G5
F4	F5	E4	G5	F3	G4	H1
H1	G3	F5	H1	H1	H1	H3

In **Table 7**, the selected optimal sensor locations for each month are presented for relative humidity. It can be observed from the Table that the selected sensors were mostly distributed along the center of the greenhouse across the months. Specifically, each month had at least five (February, March, and July) or six (April, May, June, and October) of the eight sensors distributed along the center of the greenhouse while the remaining three (3) or two (2) sensors respectively were distributed either to the right or left side of the greenhouse. This basically means that more sensors are selected from the center of the greenhouse during hotter months compared to colder ones with the exception of July which had the same number of sensors distributed in the middle as with February and March.

**Table 7:** Optimal sensor location for relative humidity.

Optimal sensor locations						
February	March	April	May	June	July	October
A1	A1	A1	B4	B6	B2	A4
A3	A4	A4	B6	B7	B7	B6
A5	B7	A5	C1	C1	C1	B7
B6	C5	B5	C2	C3	C4	C3
D6	D3	B6	C3	D3	D6	E1
E2	E1	C3	D7	D7	E2	E2
F4	E6	F6	F6	F2	E6	F5
G3	F3	H1	H1	G4	H3	H5

### 5.5 Implication of the GP-based model from a control and economic perspective

As mentioned earlier, the ultimate goal of monitoring in controlled cultivation systems is to achieve appropriate control. The advantage of the proposed framework from a control perspective is that it not only gives the optimal sensor locations for each month, but it also provides how to aggregate them efficiently to facilitate the needed control of the entire system. The symbolic representations presented in **Tables 2**, **4** for temperature and humidity respectively are the needed aggregation expressions required to obtain reference temperature and humidity that is representative of the micro-climate of the entire cultivation systems which can be fed into the control system and consequently provide control actions based on the associated control laws.

The results from the proposed model, indicate that only 8 optimally distributed sensors (less than 15% of the distributed sensors) are sufficient to facilitate efficient and effective monitoring and control of indoor environmental parameters. This reduces the entire operating cost in terms of energy use and most importantly, the cost of sensor procurement and installation can be reduced by about 75%.

## 6 Conclusions and future works

In this work, an optimal sensor location for controlled cultivation system based on Genetic Programming (GP) is proposed. Using data collected from 56 dual temperature and humidity sensors distributed within a greenhouse, reference temperature and humidity values are obtained based on the weighted average aggregation of the data. Consequently, GP is used to build symbolic models which are representative of the optimal sensors as well as how to optimally aggregate the data from the sensors. The results based on the test data shows that the reference micro-climate from the GP-based model for each month is highly correlated to those obtained based on all the 56 sensors. Furthermore based on several error metrics, it was found that the resulting error from using only 8 sensors based on the GP model is within allowable measurement error as provided from the device manufacturer which is ±2%°C.

Although, this work has been limited to only Temperature and Relative Humidity, light or Photosynthetic active radiation is another important requirement in a greenhouse or any other controlled cultivation system. Therefore in the Future, we would be interested in considering the effect of light as well as other micro-climate within the greenhouse.

## Data availability statement

The raw data supporting the conclusions of this article will be made available by the authors, without undue reservation.

## Author contributions

OA: Conceptualization, Methodology, Formal analysis, Software, Investigation, Writing - Original Draft; EA: Formal analysis, Writing - Original Draft, Writing - Review and Editing; RM: Supervision, Validation, Writing - Review and Editing; DD: Conceptualization, Data Curation; YH: Resources, Data Curation; TP: Resources, Data Curation, Supervision, Writing - Review and Editing. All authors contributed to the article and approved the submitted version.
